# Naturalist's Monthly Report

**Published:** 1811-10

**Authors:** 


### NATURALIST'S MONTHLY REPORT.

### AUGUST.

#### REAPING MONTH.

Now Ceres' gifts in waving prospect stand,

And, nodding, tempt the joyful reaper's hand.

Weather more favorable for the reaping and housing of corn than that which we have had during the present month, has I believe been seldom known. The little rain that has fallen has also been of considerable service.

The wind was westerly from the 1st to the 4th of the month, on the 5th north-west; on the 6th, 7th, and 8th, westerly; on the 9th, north-west; on the 10th and 11th north-east; on the 12th and 13th westerly; on the 14th and 15th north-east; on the 16th and 17th north-west; on the 18th and 19th south-east; on the 20th, 21st, and 22d westerly; on the 23d south-west; on the 24th southerly; on the 25th and 26th variable; from the 27th to the 29th westerly; and on the 30th and 31st north.

We had strong gales and squally weather on the 4th, 8th, and 27th, but during all the remainder of the month the weather was unusually mild and pleasant. There has been no thunder-storm.

August 2d. The farmers are beginning to house their peas and their barley.

Circular cobwebs are now observable upon the bushes and banks.

August 6th. I have lately seen several of the caterpillars of the death's-head hawk-moth (*sphynx atropos*, of Linnæus), and am informed formed that they are this year much more common than usual. Some superstitious and foolish persons have imagined that they are ominous of some evil, but they cannot even conjecture what. The moths which proceed from these are by much the largest of any of the British species, the wings of the females being frequently known to expand upwards of five inches. The caterpillars, which are of great size and extremely beautiful colour, feed on the leaves of the potatoe. During the day time they conceal themselves on the stems of plants where the leaves are large and numerous; and they feed almost wholly in the night.

August 8th. Several species of wild orache or goosefoot *(chenopodium)*, the marsh mallow, or wymote *(althæa officinalis)*, milk thistle *(carduus marianus)*, water hemp agrimony *(bidens cernua* and *bidens tripartita)*, amphibious snakeweed *(polygonum amphibium)*, and lavender thrift *(statice limonium)*, are now in flower.

August 12th. The breed of partridges is said to have suffered greatly from the wet weather that occurred about the season when young birds were hatched.

A pair of cross-bills (male and female) which were caught in the autumn of last year, have survived the winter, and are now alive and in perfect vigour at a nobleman's mansion in this neighbourhood.

August 13th. I have observed that the fruit of the hawthorn is in great abundance. The common people suppose that this is an indication of an ensuing hard winter; a notion evidently derived from the supposition that Divine Wisdom, when in severe winters it deprives the races of smaller birds of some of their usual supplies of winter's food, gives them as an equivalent an extra provision of *haws*, and other kinds of wild fruit.

August 20th. The redbreast sings.

August 22d. The clouded yellow butterflies *(papilio edusa)* are in much greater abundance than I have usually seen them. They are nearly as common as the orange tip butterflies *(papilio cardamines)* are in the spring.

In consequence of a succession of cool weather for some time past, the house flies are beginning to appear torpid and inactive.

Mushrooms are very scarce. The season has been altogether unfavorable for them.

August 26th. The black grapes begin to change colour. Peaches and nectarines are ripe; but, in this part of the country, the crop is a very unfavorable one.

August 31st. The harvest is nearly ended; and the corn will thus be housed many days earlier than has been known for several years past.

I have entirely neglected to remark the departure of the swifts. They have, however, I believe, been some time gone.

*Hampshire.*

METEORO-

